# Assessment of Fluoride Intake Risk via Infusions of Commercial Leaf Teas Available in Poland Using the Target Hazard Quotient Index Approach

**DOI:** 10.3390/foods14172944

**Published:** 2025-08-24

**Authors:** Agata Małyszek, Ireneusz Zawiślak, Michał Kulus, Adam Watras, Julia Kensy, Agnieszka Kotela, Marzena Styczyńska, Maciej Janeczek, Jacek Matys, Maciej Dobrzyński

**Affiliations:** 1 Department of Biostructure and Animal Physiology, Wrocław University of Environmental and Life Sciences, Kożuchowska 1, 51-631 Wrocław, Poland; maciej.janeczek@upwr.edu.pl; 2 Department of Human Nutrition, Wrocław University of Environmental and Life Sciences, Chełmońskiego 37/41, 51-630 Wrocław, Poland; ireneusz.zawislak@upwr.edu.pl (I.Z.); marzena.styczynska@upwr.edu.pl (M.S.); 3 Division of Ultrastructural Research, Wrocław Medical University, Chałubińskiego 6a, 50-368 Wrocław, Poland; michal.kulus@umw.edu.pl; 4 Department of Pediatric Dentistry and Preclinical Dentistry, Wrocław Medical University, Krakowska 26, 50-425 Wrocław, Poland; adam.watras@umw.edu.pl or a.watras@intibs.pl (A.W.); maciej.dobrzynski@umw.edu.pl (M.D.); 5 Institute of Low Temperatures and Structure Research, Polish Academy of Sciences, Okólna 2, 50-422 Wrocław, Poland; 6 Faculty of Dentistry, Wrocław Medical University, Krakowska 26, 50-425 Wrocław, Poland; julia.kensy@student.umw.edu.pl; 7 Medical Center of Innovation, Wrocław Medical University, Krakowska 26, 50-425 Wrocław, Poland; kotela.agnieszka@gmail.com; 8 Dental Surgery Department, Wrocław Medical University, Krakowska 26, 50-425 Wrocław, Poland

**Keywords:** dietary intake, fluoride release, health risk, mineral content, public health, tea, THQ index

## Abstract

The objective of this study was to assess the content of selected elements—fluorine, calcium and inorganic phosphorus—in infusions prepared from selected commercial leaf teas available on the Polish market. A comprehensive analysis was conducted based on tea type and geographical origin. In addition, the Target Hazard Quotient (THQ) was calculated to estimate the non-carcinogenic health risk associated with fluoride intake from tea consumption. Methods: A total of 98 leaf tea samples were analyzed, including 55 black, 27 green, 9 oolong, and 7 white teas. Standardized brewing protocols were applied. Measured parameters included pH, calcium and inorganic phosphorus content, buffer capacity, and titratable acidity. Fluoride concentrations were determined using an ion-selective electrode. Statistical analysis was performed using non-parametric methods (Kruskal–Wallis ANOVA with DSCF post hoc test), and heatmaps were generated to illustrate the distribution of THQ across different models. Results: Black teas exhibited significantly lower pH values and higher titratable acidity, buffer capacity, and inorganic phosphorus levels compared to other tea types, indicating distinct physicochemical properties. Although all THQ values for fluoride remained well below the safety threshold (THQ < 1), the highest values were observed in elderly individuals with low body weight, particularly women consuming green tea, suggesting increased vulnerability in this subgroup. Conclusions: Among the analyzed samples, black teas demonstrated the most distinct chemical profile, characterized by the lowest pH and the highest acidity, buffer capacity, and fluoride and phosphorus content—especially in teas originating from Africa and Central Asia. While fluoride exposure from leaf tea infusions does not appear to pose a direct health risk, older adults, particularly low-weight women, may be more susceptible to potential non-carcinogenic effects and should moderate their intake of high-fluoride teas.

## 1. Introduction

Tea is widely acknowledged as a notable dietary source of fluoride, largely due to the *Camellia sinensis* plant’s intrinsic capacity to accumulate fluoride from the soil and environment. During the brewing process, significant amounts of this element are readily released into the infusion. Although black tea has traditionally garnered the most scientific attention in this context, it represents only a fraction of the increasingly diverse global tea market [1]. Consumers now routinely consume a variety of non-black or “colored” teas, including green, white, and red teas (such as oolong or pu-erh), as well as a wide range of herbal infusions [2]. Black tea, made from fully oxidized leaves, is known for its strong, bold flavor and typically has the highest caffeine content. Green tea, produced by steaming or pan-firing the leaves to prevent oxidation, offers a lighter, grassy or vegetal taste and generally contains less caffeine. White tea, the least processed, uses young buds and leaves, resulting in a delicate flavor and the lowest caffeine content among true teas. Oolong tea, which is partially oxidized, falls between green and black tea in both flavor and color, offering a wide range of taste profiles [3,4,5]. These differences in processing and composition influence not only taste and caffeine content but also the amount and bioavailability of fluoride released into the brewed tea. In Poland, green tea has gained considerable popularity, driven by its perceived health benefits and broad availability, as reported by Dmowski et al. [6]. These tea types differ not only in flavor profiles and processing methods but also in their elemental composition, including the concentration, solubility, and bioavailability of fluoride [7,8].

Tea occupies a prominent place in the hydration habits of the Polish population. Recent studies indicate that hot beverages—including tea—account for approximately 45% of total fluid intake (TFI) among adults, and in some demographic groups, they are consumed even more frequently than water. The average daily intake of hot beverages is estimated at 0.73 L/day, representing nearly 30% of the recommended daily fluid intake for an adult male (2.5 L/day) [9]. Reflecting these trends, data from the Statistics Poland (GUS) indicate that the average monthly tea consumption per household member amounts to approximately 50 g [10]. Survey findings further show that around 80% of Polish respondents drink black tea—39% of them daily—while 72% report consuming green tea, though typically less often [11]. On average, individuals consume 2–3 cups of tea per day, and nearly 20% report drinking 4–5 cups daily [12]. Given that a single cup of tea can contain between 0.3 and 3.9 mg of fluoride, depending on the variety, origin, and brewing conditions- regular tea consumption may represent a significant and often underestimated source of daily fluoride intake [13,14].

Fluoride, the 13th most abundant element in the Earth’s crust, occurs naturally in the form of chemical compounds, including minerals such as fluorite, fluoroapatite, cryolite and topaz [15]. Due to its high reactivity, fluoride does not occur in a free state. The primary point of entry for this substance into the environment is through industrial emissions emanating from steelworks, coal-fired power plants, and the glass and cement industries. These emissions have the capacity to infiltrate soil, plants, and groundwater [16,17,18].

Fluoride, when administered in optimal doses, has been shown to have beneficial effects on health, primarily in terms of preventing dental caries [19,20]. It has been demonstrated that this substance strengthens enamel, thereby increasing its resistance to bacterial acids. In addition, it has been shown to support the remineralization of microdamage and to limit the carbohydrate metabolism of cariogenic bacteria, such as *Streptococcus mutans*. This, in turn, has been shown to reduce the production of acids that damage enamel [21,22,23]. Furthermore, it has been evidenced that the protective action of saliva is prolonged by this process [24,25]. Fluoride has been documented to support bone mineralization by regulating calcium and phosphate deposition. In individuals with deficiencies, it has been observed to contribute to improving bone mineral density, although its effect in this regard requires monitoring. As a trace microelement, fluoride also participates in the overall mineral homeostasis of the body, supporting the structural stability of mineralized tissues [26,27].

As is well documented, the most deleterious effects of fluoride exposure manifest during the developmental stage (including enamel fluorosis). Nevertheless, excessive consumption of this element has also been demonstrated to engender adverse health effects in adults. This phenomenon can be primarily attributed to a substantial increase in total fluoride exposure. The most significant sources of fluoride exposure include the use of oral hygiene products, the consumption of fluoridated water, supplements, certain juices and bottled waters, as well as sea fish and poultry fed with fish meal [28,29,30,31]. Furthermore, there are documented cases of occupational exposure in the chemical, metallurgical (aluminum production) and fertilizer (phosphate fertilizer production) industries [29,32].

Furthermore, excess fluoride has been demonstrated to exert a nephrotoxic effect, resulting in a deterioration of kidney filtration function, and disrupting cellular metabolism by inhibiting glycolytic enzyme activity [33,34]. Fluoride has been evidenced to interfere with the process of iodine uptake by thyroid cells, thereby weakening the synthesis of T3 and T4 hormones. It is an established fact that long-term exposure to elevated concentrations of fluoride has been shown to result in hypothyroidism, a condition that is characterized by a range of symptoms, including fatigue, weight gain, dry skin and low mood [35,36]. Consequently, the long-term accumulation of fluoride in the bones results in skeletal fluorosis (*fluorosis ossium*). Fluoride replaces hydroxyl groups in hydroxyapatite, forming fluoroapatites, which leads to increased density but reduced bone elasticity [37]. The clinical symptoms primarily manifest as bone and joint pain, stiffness and thickening of the bones, as well as increased fragility [38,39].

The Target Hazard Quotient (THQ) represents a widely accepted methodology for assessing non-carcinogenic health risks associated with chronic exposure to potentially harmful substances through dietary intake [40]. This risk assessment tool quantifies the ratio between estimated chronic daily intake (CDI) and the established oral reference dose (RfD) for a specific compound, with values below 1.0 indicating negligible risk and values exceeding 1.0 suggesting potential non-carcinogenic health effects [41]. For this investigation, the conventional THQ methodology has been modified to consider fluoride content from both tea leaves and tap water used for preparation, providing a comprehensive assessment of total fluoride exposure through tea consumption. This enhanced approach provides a more comprehensive assessment of total fluoride exposure through tea consumption, taking into account the dual sources of fluoride in the final infusion [42]. The modified CDI calculation considers multiple exposure parameters including daily consumption patterns derived from GUS data showing tea infusion intake ranging from 0.132 L/day for adults aged 19–59 years to 0.197 L/day for individuals ≥ 75 years, exposure frequency and duration, and body weight variations [43]. By stratifying the analysis across age groups and gender categories using reference body weights from Polish nutritional standards and incorporating local water fluoride concentrations (0.22 mg/L for Wrocław) [44]. This approach enables population-specific risk characterization with geographical relevance.

Given the concerning potential of tea to contribute substantially to daily fluoride intake, further investigation into its elemental composition is essential. The systematic review conducted by our team showed that not only the tea type but also brewing parameters including infusion time, leaf fragmentation, water fluoride content, and temperature significantly influence the fluoride released into the beverage [1]. The previous study, focusing only on black tea presented that its consumption may provide fluoride levels approaching or even exceed recommended limits, particularly with frequent consumption [45]. However, black tea represents just one segment of a much broader and continuously expanding global tea market. In recent years, consumer preferences have increasingly diversified, with growing interest not only in traditional black teas but also in a wider selection of so-called “colored” teas such as green, white, and red teas, including varieties like oolong and pu-erh which differ in processing methods, oxidation levels, and chemical composition [3,7]. Additionally, herbal infusions made from non–*Camellia sinensis* plants, such as chamomile, rooibos, peppermint, or hibiscus, have gained substantial popularity as caffeine-free alternatives [46,47]. This ongoing shift in consumer behavior has introduced a wide range of beverage products with potentially varying fluoride contents. As a result, there is a pressing need to better understand and compare the fluoride levels present across these diverse tea and infusion types in order to assess their contribution to total fluoride intake more accurately.

This study is a direct continuation of our latest study and aims to evaluate and compare the concentrations of fluoride and other elemental components in leaf white, green, and red teas (oolong), in relation to black tea, which was the focus of our prior investigation [45]. Understanding the elemental composition of these teas is critical given the health concerns associated with excessive fluoride intake. By extending our research scope beyond black tea, this study provides important insights into the variability of fluoride and elemental content among different tea types, contributing to better consumer risk assessment and safety recommendations.

## 2. Materials and Methods

### 2.1. Research Groups

The authors conducted a comprehensive study, evaluating teas from various manufacturers. All items were acquired from retail stores located in Wrocław. The present study focused on leaf teas (*n* = 98). In order to establish a clear framework for analysis, the tea under scrutiny was traced back to its geographical origins, which were divided into three distinct regions. The following regions were included in the study: Central Asia (*n* = 49), East Asia (*n* = 41) and Africa (*n* = 8). The details are provided in Table 1.

### 2.2. Methodology for Preparing Tea Infusions

A quantity of 2.0 g of leaf tea was subjected to a brewing process for a duration of three minutes, with the tea being covered during this time. A quantity of 200 mL of commercial deionised water (EUROMEX Sp. z o. o., Szczucin, Poland) was utilised in the experiment. The temperature employed corresponded to the manufacturer’s recommended range of 95–100 °C. The temperature of the water used for the brewing process was strictly regulated by way of an electric kettle (Amica model KM6011, Wronki, Poland). The utilisation of deionised water in the brewing of tea followed a comparable procedure. Water quality was monitored with a TDS (total dissolved substances) meter (TDS-3, ABC-RC, Wieprz, Poland). 7 mg/L—this was the TDS value for the deionised water used in the study. The obtained infusion was subsequently filtered through filter paper. This action yielded a transparent brew. Laboratory analysis of the solutions was conducted only after the infusion had reached room temperature. In order to ensure the validity of the results, each analysed parameter was measured at three separate times.

### 2.3. The Measurement of the pH Value of Tea Infusions

The tea infusions were prepared fresh each time. The measurements in question were carried out at room temperature using a pH electrode supplied by Eurosensor (Eurosensor, Gliwice, Poland) to determine the pH value. The device was connected to a CPI-505 pH/ion meter (Elmetron, Zabrze, Poland). The experiments were carried out using reagents that were very pure. The beverages were made using deionised water, as explained earlier.

### 2.4. Procedure for Measurement of the Buffering Capacity of Tea Infusions

The term ‘buffer capacity’ is employed to denote the ability to counteract the effect of the introduction of a strong base or strong acid on the pH of the buffer solution (i.e., the capacity to bind H^+^ and OH^−^ ions) [45]. The buffering capacity was determined by the authors through the addition of 0.1 M HCl (reagent purity, Chempur, Poland) to the infusion. The calculation was performed in accordance with the formula delineated below:

$$Buffer\;capacity\;(\frac{mol}{L})=(\frac{0.01}{V})\times (pH_{2}-pH_{1})$$

where

- *pH*_1_ is the pH of the brewed tea;
- *pH*_2_ is the resultant pH value subsequent to the addition of 0.1 M HCl.

### 2.5. Titratable Acidity of Tea Infusions Determination

Titratable acidity is stated in millimoles per litre (mmol/L) and refers to the volume of 0.1 M NaOH (reagent purity, Chempur, Poland) needed to neutralise the solution’s acidic components to a pH of 7.

### 2.6. The Method for the Determination of Calcium and Inorganic Phosphorus in Tea Infusions

The concentration of inorganic phosphorus was determined using the phosphomolybdate method at 340 nm, with deionised water (TDS = 7 mg/L, EUROMEX Sp. z o.o., Szczucin, Poland) as a standard. This method is based on forming a phosphomolybdate complex in an acidic medium, which is then reduced to a measurable molybdenum blue complex using a spectrophotometer. The chemicals employed in this study included detergents, ammonium molybdate and sulphuric acid (reagent grade, Spinreact Kit, Spinreact, S.A./S.A.U., Sant Esteve de Bas, Spain).

The quantification of calcium was conducted through the utilisation of the Arsenazo III method, employing a wavelength of 630 nm. Deionised water (TDS = 7 mg/L, EUROMEX Sp. z o. o., Szczucin, Poland) was utilised as a reference standard. The method is grounded in the process of forming a stable purple complex between calcium ions and arsenazo III in imidazole buffer (pH 6.5) (reagent grade, Spinreact Kit, Spinreact, S.A./S.A.U., Sant Esteve de Bas, Spain).

### 2.7. The Method for the Quantitation of Fluoride in Tea Infusions

The determination of fluoride was conducted by employing an ORION 9609 ion-selective electrode (Thermo Fisher Scientific, Waltham, MA, USA), in strict accordance with the manufacturer’s guidelines, in conjunction with a CPI-551 ELMETRON pH/ionometer microcomputer (Elmetron, Zabrze, Poland). This electrode is of a combined nature. Consequently, the utilisation of a separate reference electrode is rendered redundant for the experimental procedure.

### 2.8. The Health Risk Assessment

The health risk related to the potential non-carcinogenic effect of fluoride intake from tea infusion was assessed using the Target Hazard Quotient (*THQ*), calculated according to the following formula [48]:

$$THQ=\frac{CDI}{RfD}$$

the chronic daily intake (*CDI*) is the estimated fluoride intake from tea infusion (mg/kg body weight/day), while the oral reference dose (*RfD)* for fluoride in adults, established by the U.S. Environmental Protection Agency (EPA), is 0.05 mg/kg body weight/day (2). A *THQ* value greater than 1 indicates a significant potential non-carcinogenic effect on health, while a value below 1 indicates no effect.

The *CDI* was estimated based on the formula:

$$CDI=\frac{C\times IR\times EF\times ED}{BW\times AT}$$

where *C* is the total fluoride content in the tea infusion (mg/L), *IR* is the average daily volume of tea infusion consumed (L/day), *EF* is the frequency (days/year) and *ED* is exposure duration (years), *BW* is body weight (kg) and *AT* is the total period over which the exposure is averaged (days). For non-carcinogenic risk assessment, the averaging time (AT) was assumed to be equal to the exposure duration multiplied by 365. Health risk assessments were conducted for the adult Polish population, stratified by gender and age, with body weight variations taken into account (Table 2). Estimates of average tea infusion consumption were based on data from Statistics Poland (GUS), as shown in Table 3 [43].

The total fluoride content in the tea infusion was calculated using the formula:

$$C=C_{F}+T_{F}$$

where *C_F_* is the concentration of fluoride in the tea infusion prepared using deionized water (mg/L), *T_F_* is the concentration of fluoride in the tap water (mg/L).

### 2.9. Statistical Analysis

The statistical analysis carried out in this current study centred on the identification of potential differences between various types of tea (black, green, oolong, white) and their respective regions of origin (Central Asia, East Asia, and Africa), as well as exploring potential correlations between the variables that were examined. Since most of the analyzed variables did not represent a normal distribution, the current study used a nonparametric Kruskal–Wallis ANOVA test to detect differences in the distribution of distinct variables among tea types. The post hoc DSCF test was then used for further pairwise comparisons. It is important to note that the statistical analysis was conducted using Jamovi software version 2.6 (Jamovi, Sydney, Australia) and the R Statistical Environment (R Core Team, Vienna, Austria). Heatmaps depicting the distribution of THQ for different models were created in the R statistical environment using the ggplot2 [3] and viridis [4] packages.

## 3. Results

### 3.1. Physicochemical Properties of Teas

**Figure 1:**
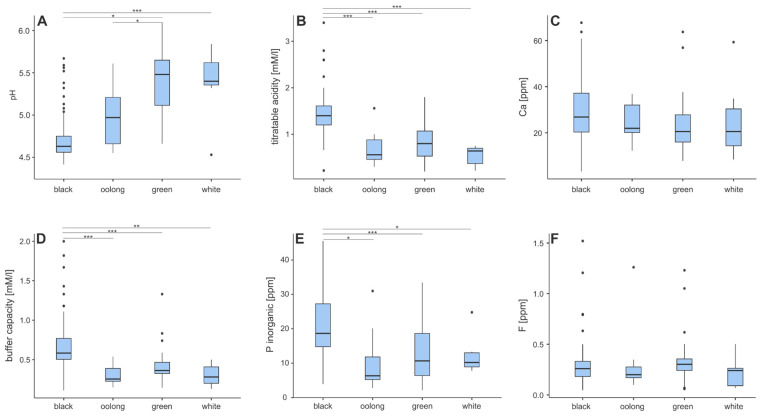
Box-plots representing differences among tea types. (**A**) pH; (**B**) titratable acidity; (**C**) Ca; (**D**) buffer capacity; (**E**) inorganic phosphorus, (**F**) Generally, black teas tend to stand out significantly from other tea types. Each box shows the interquartile range (IQR), with the median marked by a bold line in the middle. Whiskers show the range within 1.5 IQR and dots represent outliers. Each presented variable obtained a *p*-value of less than 0.001 in the Kruskal–Wallis ANOVA test. The results of the post hoc test are marked with asterisks. *—*p* < 0.05; **—*p* < 0.01; ***—*p* < 0.001.

Statistically significant differences in physicochemical properties among the analyzed tea types are presented in Figure 1.

Black teas exhibited a significantly lower pH (median = 4.63) compared to oolong (4.97), green (5.48), and white (5.40) teas (*p* < 0.001). Among non-black teas, oolong tea showed a significantly lower pH than green tea (*p* < 0.05), while the difference between green and white teas was not statistically significant (Figure 1A). Regarding titratable acidity, black teas had markedly higher values (median = 1.40 mM/L) compared to oolong (0.56 mM/L), green (0.80 mM/L), and white (0.64 mM/L) teas, with differences highly significant across all comparisons (*p* < 0.001; Figure 1B).

Buffer capacity was also highest in black teas (0.58 mM/L), significantly exceeding values in oolong (0.26 mM/L), green (0.36 mM/L), and white (0.30 mM/L) teas (Figure 1D), with all differences being statistically significant (*p* < 0.001 or *p* < 0.01). Inorganic phosphorus content followed a similar trend: black teas had significantly higher concentrations (18.7 ppm) than oolong (6.35 ppm), green (10.7 ppm), and white (10.2 ppm) teas (Figure 1E). The differences between black tea and all other types were statistically significant (*p* < 0.05 to *p* < 0.001). In contrast, calcium content (Ca) and fluoride content (F) did not show statistically significant differences between the tea types (Figure 1C,F). Although numerical differences were observed—with black teas having slightly higher median Ca levels and fluoride concentrations—these variations did not reach statistical significance.

Detailed descriptive statistics, including medians, interquartile ranges, and outliers, are provided in Supplementary Tables S1 and S2.

### 3.2. Chemical Composition of Teas Across Different Regions

**Figure 2:**
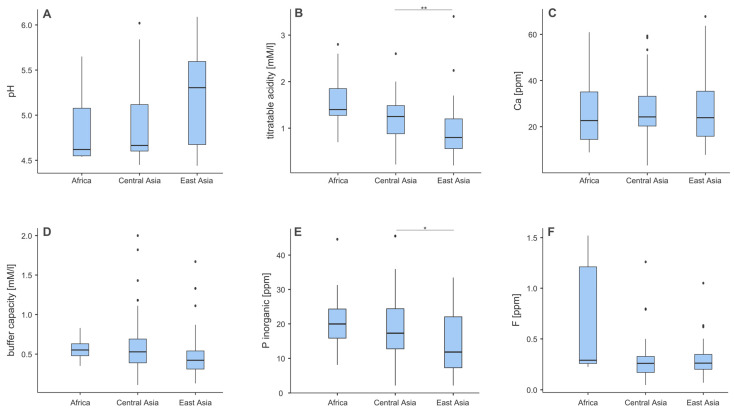
Box-plots representing differences depending on origin. types. (**A**) pH; (**B**) titratable acidity; (**C**) Ca; (**D**) buffer capacity; (**E**) inorganic phosphorus, (**F**) Each box shows the interquartile range (IQR), with the median marked by a bold line in the middle. Whiskers show the range within 1.5 IQR and dots represent outliers The *p*-values obtained from the Kruskal–Wallis ANOVA test are 0.022 and 0.001 for plots A and B, respectively. The results of the post hoc test are marked with asterisks. *—*p* < 0.05; **—*p* < 0.01.

Figure 2 illustrates regional differences in the chemical composition of tea infusions. Among the six measured variables, statistically significant differences were observed for titratable acidity (Figure 2B) and inorganic phosphorus content (Figure 2E).

Teas from Central Asia had significantly higher titratable acidity (median: 1.25 mM/L) and inorganic phosphorus levels (17.3 ppm) compared to teas from East Asia (0.80 mM/L and 11.7 ppm, respectively). Similarly, African teas showed the highest values overall for both parameters, with a median titratable acidity of 1.40 mM/L and inorganic phosphorus content of 20.0 ppm. However, these differences reached statistical significance primarily in comparisons with East Asian teas.

For the remaining variables—pH, calcium (Ca), buffer capacity, and fluoride (F)—no statistically significant regional differences were observed, although some numerical variation was evident (Figure 2A,C,D,F). The median pH values were as follows: Africa—4.62, Central Asia—4.67, and East Asia—5.30 (Figure 2A), suggesting a trend toward higher pH in East Asian teas. Calcium content was relatively consistent across regions, ranging from 22.6 ppm (Africa) to 24.2 ppm (Central Asia) (Figure 2C). Buffer capacity was slightly higher in African teas (0.55 mM/L) and lowest in East Asian teas (0.42 mM/L) (Figure 2D). Notably, fluoride concentrations were highest in African teas (0.29 ppm), followed by East Asia (0.27 ppm) and Central Asia (0.26 ppm), although these differences were not statistically significant (Figure 2F).

### 3.3. Target Hazard Quotient Analysis

**Figure 3:**
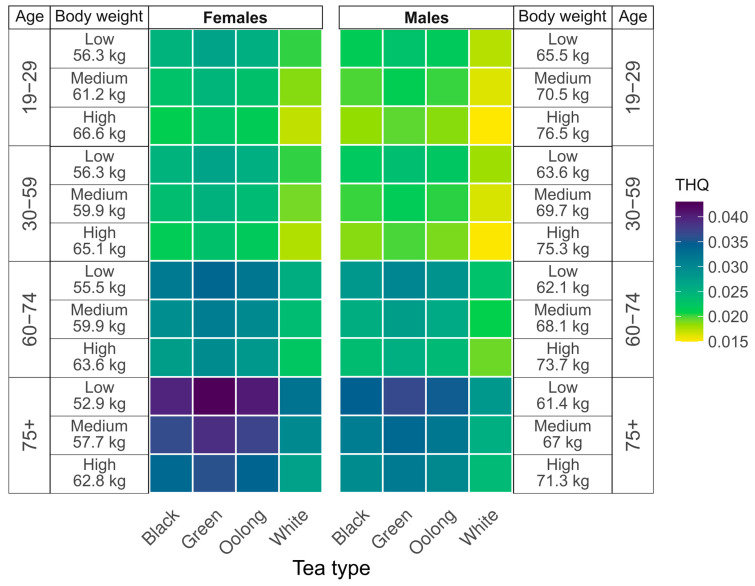
Heat maps of Target Hazard Quotient (THQ) for fluoride in tea infusions, based on models considering age (in years), gender and reference body weight (BMI = 22 kg/m^2^), the low body weight corresponds to the 10th percentile and the high to the 90th percentile.

Assessment of non-carcinogenic health risk related to fluoride intake with tea infusion are presented in Figure 3. The highest non-carcinogenic health risk associated with fluoride intake from tea infusions was observed in women aged 75 years and older. In this group, the Target Hazard Quotient (THQ) for women with low reference body weight was highest for green tea (0.043) and lowest for white tea (0.033). For black and oolong teas, THQ values ranged from 0.040 to 0.041. In contrast, the lowest THQ values were recorded in women aged 19–29 years with reference body weight at the 90th percentile. In this subgroup, THQ values were as follows: 0.017 for white tea, 0.022 for oolong tea, 0.023 for green tea, and 0.021 for black tea. A similar pattern was observed among men. The highest THQ was found in men aged 75+ years with low reference body weight who consumed green tea (0.037). The lowest value was observed in men aged 19–29 years at the 90th percentile of reference body weight, who consumed white tea (0.015). Overall, women exhibited higher THQ values than men, and older individuals were generally more susceptible to non-carcinogenic health risks associated with fluoride intake from tea infusions compared to younger individuals.

### 3.4. THQ Calculator

Based on the Target Hazard Quotient (THQ) formula, a calculator has been developed to estimate the non-carcinogenic health risk associated with oral fluoride intake from tea, considering the fluoride content of the tap water used to prepare it. Based on data entered by the user, such as body weight, average daily tea consumption (expressed in number of 200 mL cups), selection of a specific type of tea from a list or individual fluoride content in the infusion (ppm), and fluoride content in tap water (based on data provided by the local water supplier), the calculator calculates the THQ value and informs the user whether there is a non-carcinogenic health risk associated with oral consumption of fluoride with tea infusion (THQ ≥ 1) or not (THQ < 1) (see ‘Supplementary Materials: THQ Calculator’).

## 4. Discussion

This study aimed to comprehensively evaluate the fluoride content and associated health risks across different types of colored leaf teas, extending beyond our previous investigation of black tea to include green, white, and oolong varieties from diverse geographical origins. Our results demonstrate that while significant physicochemical differences exist between tea types—particularly the distinct acidic profile of black tea compared to other varieties in our earlier research—fluoride and calcium concentrations showed no statistically significant variation among colored tea types or geographical regions in the present study. These findings provide an important contrast to our previous black tea investigation [45], where we observed substantial differences in fluoride content based on tea form, with tea bags showing significantly higher fluoride levels (mean 0.74 mg/L, maximum 1.82 mg/L) compared to the current study’s colored leaf teas (ranging 0.285–0.659 mg/L across regions). The relatively consistent fluoride levels across colored tea types support the hypothesis that fluoride content in leaf teas is largely determined by environmental factors during cultivation rather than post-harvest processing techniques. However, when compared to our previous findings on black tea, processing methods and tea form significantly influenced fluoride bioavailability in commercial black tea products forms [45]. This differentiation has important implications for public health assessments, as it suggests that while consumers switching between different colored leaf tea types may not significantly alter their fluoride exposure, the choice between colored leaf teas and black tea preparations (particularly black tea bags) represents a more critical decision for managing total dietary fluoride intake [1].

The observed physicochemical differences among tea types, particularly between green, white and oolong teas compared to black tea reflect the varying degrees of oxidation and fermentation processes these teas undergo, which profoundly influence their chemical profiles [1]. Black tea consistently demonstrates a significantly lower pH than the other tea types, accompanied by higher titratable acidity, buffer capacity, and inorganic phosphorus content, marking its distinct acidic profile relative to less processed teas. This heightened acidity in black tea results from extensive enzymatic oxidation during fermentation, which promotes the formation of a range of organic acids, including gallic, chlorogenic, and oxalic acids, known to contribute to lower pH and increased titratable acidity [50]. The amplified buffer capacity observed in black tea reflects a greater concentration of acid-base modifiers capable of stabilizing pH changes, potentially influencing its sensory attributes such as bitterness and astringency [51]. In contrast, green, white, and oolong teas, which experience partial or minimal oxidation, maintain relatively higher pH values and display lower titratable acidity and buffer capacity, corresponding to milder acid profiles. The unique status of oolong tea is notable; it exhibits a significantly lower pH than green tea, despite similarities in other parameters, likely due to its intermediate fermentation level, which partially oxidizes phenolic compounds and affects acidity [50,52]. Furthermore, the significantly lower inorganic phosphorus content in green, white, and oolong teas compared to black tea may reflect reduced formation of polyphenol-phosphorus complexes, which tend to increase during the oxidation process in black tea [53]. These phosphorus complexes could play a role in modulating mineral bioavailability and influencing the nutritional qualities of teas. Overall, the physicochemical distinctions evidenced by these parameters underscore the critical role of processing methods in shaping tea chemistry, sensory qualities, and potential health-related effects.

Despite expectations that geographic origin would significantly affect mineral content due to varying soil composition and environmental conditions, the analysis showed that fluoride and calcium levels showed no statistically significant differences between Central Asia, East Asia and Africa. The observed numerical differences—with African teas showing the highest fluoride levels (0.29 mg/L) and Central Asian teas the lowest (0.26 mg/L)—while potentially significant from a public health perspective, did not reach statistical significance. This lack of regional significance for fluoride content contradicts several previous studies that found significant geographic differences in fluoride levels in tea, particularly between teas of Asian and African origin [54]. Consistent calcium levels across regions (22.6–24.2 mg/L) suggest that calcium availability in tea infusions is relatively stable regardless of growing location, which may indicate similar bioavailability patterns in different soil types [55,56]. These findings challenge the assumption that geographic origin serves as a reliable predictor of mineral content in tea infusions and highlight the complex interaction between soil chemistry, climate and agricultural practices that ultimately determine the elemental composition in the final product.

Adults constitute the primary demographic of consumers of loose-leaf teas, primarily due to their appreciation of the quality, taste and health benefits associated with this type of tea. The consumption of leaf teas, including green, black, and pu-erh varieties, remains comparatively low among children in developed countries. However, it is not recommended to consume these infusions regularly due to their chemical composition. These teas contain caffeine (theine), which has been demonstrated to exert an adverse effect on the central nervous system of children, manifesting in symptoms such as agitation, sleep disturbances, difficulty concentrating, and changes in blood pressure [57]. Furthermore, the tannins and polyphenols present in tea leaves possess anti-nutritional properties, thereby limiting the absorption of iron, calcium, and other micronutrients. This may potentially increase the risk of developing anaemia and mineralization disorders [58,59].

In practice, children most often consume water and fruit or herbal teas (e.g., chamomile, fennel), as well as ready-made drinks such as iced tea, which are often sweetened and do not contain classic tea leaf extracts. Research undertaken in Mexico has indicated an association between the ingestion of fluoride, as found in ready-made sweetened beverages and drinking water, and a potential health risk to paediatric patients. Rocha-Amador et al. demonstrated that 73.2% of beverages (out of 82 tested) exceeded the acceptable fluoride standards [60]. The study concluded that the highest fluoride content was found in cheaper products. This finding suggests the use of substandard water. Consequently, Pérez-Vázquez et al. conducted a study on children residing in three zones (metallurgical, agricultural and industrial) of the city of San Luis Potosí (Mexico), located in a region affect-ed by endemic hydrofluorosis [61]. In the cohort of children residing within the metallurgical zone, the HQ (Hazard Quotient) index was determined to be 1.4, signifying a substantial health hazard associated with the ingestion of fluoride present in the local potable water. In contrast, in the other urban zones—agricultural and industrial—the HQ value was lower, but still significant. Thus, the ingestion of sweetened beverages with elevated fluoride levels has the potential to result in dental fluorosis in children, particularly when concomitant with exposure to fluoride from drinking water or oral hygiene products. The necessity for preventive measures, including water purification and public education, is emphasized.

The Target Hazard Quotient analysis [40] showed that all of the tea types studied pose minimal non-carcinogenic health risks under typical consumption patterns, with THQ values ranging from 0.015 to 0.043—well below the hazard threshold (THQ > 1.0). In addition, other studies on various elements, including toxic metals, have reported a low non-carcinogenic health risk for European consumers of black and green tea from European plantations [62,63,64]. Calculations indicate that a person weighing 70 kg would need to consume about 27 cups of colored leaf tea per day to approach potentially harmful levels of fluoride exposure (THQ > 1), showing a significant margin of safety with normal consumption patterns. However, these results should be compared with previous studies on black tea, in which consumption of three cups per day of certain African black tea bags can approach or exceed THQ > 1, especially in lower-weight individuals. This striking difference is directly supported by the fluoride concentrations observed in our current study compared to the previous black tea study: while colored leaf teas showed fluoride levels of 0.26–0.29 mg/L, black tea bags showed much higher concentrations reaching 1.82 mg/L, with African-origin tea bags showing average fluoride levels of 0.89 mg/L compared to 0.32–0.40 mg/L in leaf teas from the same regions. The lower risk of colored leaf teas compared to black tea bags can be attributed to differences in processing methods, leaf maturity, particle size affecting extraction efficiency, and quality grading systems that tend to reserve younger, higher quality leaves for bulk products. Women 75 years of age and older with lower body weights were the demographic group at highest risk in our analysis, underscoring the importance of considering individual physiological factors in assessing fluoride risk along with tea form and origin when developing consumption guidelines.

Calculations indicate that an average adult weighing 70 kg would have to consume 61 cups (200 mL each) of properly prepared green leaf tea—characterised by the lowest fluoride content among the samples tested—or 35 cups of leaf oolong tea, which had the highest concentration of this element in this study, to exceed the acceptable daily intake of fluoride considered potentially harmful to health. For comparison, consuming just 22 cups of black tea bags per day may be associated with a risk of noncancerous health effects related to excessive fluoride exposure [45]. The form, type of tea and method of preparation all have a significant impact on fluoride content. Tea bags generally have a higher fluoride concentration, which may be related to the finer grinding of the leaves and thus a larger surface area for the release of the element into the solution [1]. In addition, it is crucial to adhere to the recommended brewing time. Extending the brewing time significantly increases the amount of fluoride in the infusion. For example, an infusion of oolong leaf tea contained 0.92 mg F/L after 3 min, 1.16 mg F/L after 5 min, 1.31 mg F/L after 10 min, and as much as 1.76 mg F/L after 120 min of brewing. Importantly, not only brewing time but also temperature represents a critical factor influencing mineral extraction—previous studies [64] demonstrated that higher infusion temperatures significantly enhance the mobilization of elements from tea leaves into the infusion. Depending on the brewing time, the number of cups of infusion that would exceed the acceptable daily intake of fluoride was 15 (3 min), 13 (5 min), 12 (10 min) and 9 (120 min) cups per day, respectively [65]. It is worth noting that tea infusion represents only one of several dietary sources of fluoride, and total exposure should be considered in the context of combined intake from drinking water, other foods, and dental products [15,66,67,68,69,70,71,72,73].

In interpreting these results, several limitations must be considered, including the use of deionized water for tea preparation, which can affect brewing conditions where tap water contains additional fluoride and minerals that can affect both extraction efficiency and total fluoride exposure [74]. The standardized brewing protocol (2 g of tea per 200 mL of water for 3 min at 95–100 °C) may not account for the full range of consumer tea preparation methods that may affect the release of fluoride from tea leaves. Future studies should investigate the interaction between water hardness and fluoride extraction, examine the effects of different brewing parameters on mineral release, and increase the sample size to include more diverse tea varieties and origins. It would be particularly important to study colored tea bags using the same methodological approach as our previous study of black tea, to determine whether the processing effects observed in black tea bags extend to other types of tea. Long-term epidemiological studies tracking actual consumption patterns and biomarkers of fluoride exposure would provide valuable validation of these in vitro risk assessments. Moreover, investigating the mechanisms underlying the apparent differences between the bioavailability of fluoride from leaf tea and tea bags could inform both consumer guidance and regulatory standards.

## 5. Conclusions

Among the leaf teas that were investigated, black teas were found to possess significantly divergent physicochemical properties compared to the other teas. These properties included the lowest pH, the highest titratable acidity and buffer capacity, and the highest inorganic phosphorus content. This may contribute to greater bioavailability of fluoride and other elements. A comparative analysis of teas from Africa and Central Asia with those from East Asia revealed that the former contained higher levels of phosphorus and exhibited greater acidity. The highest fluoride concentrations were detected in African tea infusions. The study indicates that the geographical origin of leaf teas has a significant impact on their fluoride and phosphorus content, as well as titration acidity. All Target Hazard Quotient (THQ) values for fluoride were found to be below one, indicating that no direct health risk is posed by the consumption of the leaf teas under study. The highest THQ values (0.043) were observed in individuals aged 75 and over with low body weight, particularly in women who consumed green tea. However, research has identified a heightened vulnerability to the non-carcinogenic consequences of fluoride intake from tea among female populations and the elderly. It is recommended that individuals belonging to these groups exercise caution when consuming teas with higher fluoride content.

## Figures and Tables

**Table 1:** Specification of investigated leaf teas.

No	Brand	Tea Name	Type	Country	Region
1	Ahmad Tea	Assam Tea	black	India	Central Asia
2	Ahmad Tea	Ceylon Tea	black	UAE	Africa
3	Ahmad Tea	English Breakfast	black	UAE	Africa
4	Ahmad Tea	Green Tea	green	UAE	Africa
5	Ahmad Tea	Jasmine Green Tea	green	UAE	Africa
6	Akbar	Earl Grey	black	Sri Lanka	Central Asia
7	Akbar	Pure Ceylon Tea	black	India	Central Asia
8	Astra	Safari	black	Kenya	Africa
9	Big-Active	Ceylon	black	UAE	Africa
10	Big-Active	Pure Green	green	China	East Asia
11	Chelton	English Green Tea	green	Sri Lanka	Central Asia
12	Chelton	Gunpowder Tea	green	Sri Lanka	Central Asia
13	Dilmah	Ceylon Gold	black	Sri Lanka	Central Asia
14	Dilmah	Ceylon Premium Tea	black	Sri Lanka	Central Asia
15	Dilmah	Ceylon Supreme	black	Sri Lanka	Central Asia
16	Dilmah	English Breakfast	black	Sri Lanka	Central Asia
17	Dilmah	Gourmet Earl Grey tea	black	Sri Lanka	Central Asia
18	Eternal	Finest Top	black	Sri Lanka	Central Asia
19	Five o’clock	Assam Halmari GTGFBOP	black	India	Central Asia
20	Five o’clock	Assam Halmari GTGFOP1CL	black	India	Central Asia
21	Five o’clock	Assam Satrupa	black	India	Central Asia
22	Five o’clock	Assam Singlijan	black	India	Central Asia
23	Five o’clock	Assam Tonganagaon	black	India	Central Asia
24	Five o’clock	Ceylon Ahinsa	black	Sri Lanka	Central Asia
25	Five o’clock	Ceylon Lumbini	black	India	Central Asia
26	Five o’clock	Ceylon Vithanakande	black	Taiwan	East Asia
27	Five o’clock	China Wild Buds	white	China	East Asia
28	Five o’clock	China Moonlight White	white	China	East Asia
29	Five o’clock	China Oolong Ti Kuan Yin	oolong	China	East Asia
30	Five o’clock	China Panyong Golden Needle	black	China	East Asia
31	Five o’clock	China White Monkey	white	China	East Asia
32	Five o’clock	China Wuyi Rou Gui	oolong	China	East Asia
33	Five o’clock	Darjeeling Flower Balasun	black	India	Central Asia
34	Five o’clock	Darjeeling Gielle	black	India	Central Asia
35	Five o’clock	Darjeeling Liza Hill DJ5/21	black	India	Central Asia
36	Five o’clock	Darjeeling Musk Puttabang	black	India	Central Asia
37	Five o’clock	Darjeeling Nagr DJ2	black	India	Central Asia
38	Five o’clock	Darjeeling Shree Dwarika	black	India	Central Asia
39	Five o’clock	Darjeeling Teesta Valley DJ11	black	India	Central Asia
40	Five o’clock	Formosa Finest Oolong	oolong	Taiwan	East Asia
41	Five o’clock	Formosa Lapsang Souchong	black	Taiwan	East Asia
42	Five o’clock	Formosa Oolong Dung Ting	oolong	Taiwan	East Asia
43	Five o’clock	Formosa Oolong High Mountain	oolong	Sri Lanka	Central Asia
44	Five o’clock	Formosa Pi Lo Chun	green	China	East Asia
45	Five o’clock	Formosa Supreme Gancy Oolong	oolong	Taiwan	East Asia
46	Five o’clock	Golden Yunan	black	China	East Asia
47	Five o’clock	Golden Yunan Superior	black	China	East Asia
48	Five o’clock	Gunpowder Temple of Heaven	green	China	East Asia
49	Five o’clock	Japan Bancha Tenryu	green	Japan	East Asia
50	Five o’clock	Japan Black Tea	black	Japan	East Asia
51	Five o’clock	Japan Gabalong	green	Japan	East Asia
52	Five o’clock	Japan Gyokuro Tohei	green	Japan	East Asia
53	Five o’clock	Japan Gyokuro Uji	green	Japan	East Asia
54	Five o’clock	Japan Kabuse Kagashima	green	Japan	East Asia
55	Five o’clock	Japan Sencha Fukujyu	green	Japan	East Asia
56	Five o’clock	Japan Sencha Shizuoka	green	Japan	East Asia
57	Five o’clock	Keemun	black	China	East Asia
58	Five o’clock	Nilgiri Platinum Needles	white	India	Central Asia
59	Five o’clock	Oolong Sun Moon Lake Ruby	oolong	Taiwan	East Asia
60	Five o’clock	Pai Mu Tan Superior	white	China	East Asia
61	Five o’clock	South India Nilgiri Bamboo	green	India	Central Asia
62	Five o’clock	South India Nilgiri Kukicha Roasted	black	India	Central Asia
63	Five o’clock	South India Nilgiri Long Jing	green	India	Central Asia
64	Five o’clock	South India Nilgiri Slender	green	India	Central Asia
65	Five o’clock	South India Nilgiri White Tea Peony	white	India	Central Asia
66	Five o’clock	South India Nilgiri Wulong	oolong	India	Central Asia
67	Five o’clock	Yunan Special Green	green	China	East Asia
68	Five o’clock	Yunnan Green Superior	green	China	East Asia
69	HAYB	Cui Min Spring	white	China	East Asia
70	HAYB	Oolong Formosa	oolong	Taiwan	East Asia
71	HAYB	Sencha	green	China	East Asia
72	Impra Tea	Royal Elixir Tea (green packaging)	green	Sri Lanka	Central Asia
73	Lipton	Yellow Label	black	Kenya	Africa
74	Lord Nelson	Green	green	China	East Asia
75	Lord Nelson	Assam	black	India	Central Asia
76	Lord Nelson	Earl Grey with Lemon Peel	black	China	East Asia
77	Loyd	Earl Grey	black	Sri Lanka	Central Asia
78	Loyd	Green	green	China	East Asia
79	Loyd	Madras	black	India	Central Asia
80	Loyd	Yunnan	black	China	East Asia
81	Remsey	Green Pure Leaf	green	Kenya	Africa
82	Sir Adalbert’s Tea	Black Tropical Tea	black	Sri Lanka	Central Asia
83	Sir Adalbert’s Tea	Earl Grey	black	Sri Lanka	Central Asia
84	Vintage Teas	Dimbula	black	Sri Lanka	Central Asia
85	Vintage Teas	Earl Grey	black	Sri Lanka	Central Asia
86	Vintage Teas	Kandy	black	Sri Lanka	Central Asia
87	Vintage Teas	Natural Green Tea	green	Sri Lanka	Central Asia
88	Vintage Teas	Nuwara Eliya	black	Sri Lanka	Central Asia
89	Vintage Teas	Organic Black Tea	black	Sri Lanka	Central Asia
90	Vintage Teas	Organic Green Tea	green	Sri Lanka	Central Asia
91	Vintage Teas	Ruhuna	black	Sri Lanka	Central Asia
92	Vintage Teas	Sabaragamuwa	black	Sri Lanka	Central Asia
93	Yunnan	Black Tea	black	China	East Asia
94	Yunnan	Green Tea	green	China	East Asia
95	ZAS	Assam	black	India	Central Asia
96	ZAS	Madras	black	India	Central Asia
97	ZAS	Yunnan	black	China	East Asia
98	ZAS	Yunan Black Tea	black	China	East Asia

**Table 2:** Reference body weight by age, according to Rychlik et al. [49].

Gender	Age (Years)	Reference Body Weight (kg) *		
		10th Percentile	Median	90th Percentile
Men	19–29	65.5	70.5	76.5
	30–59	63.6	69.7	75.3
	60–74	62.1	68.1	73.7
	≥75	61.4	67	71.3
Women	19–29	56.3	61.2	66.6
	30–59	56.3	59.9	65.1
	60–74	55.5	59.9	63.6
	≥75	52.9	57.7	62.8

* The reference body weight for a given height, calculated based on a BMI of 22 kg/m^2^.

**Table 3:** Estimated average tea infusion intake, according to GUS [43].

Age (Years)	Annual Tea Consumption (g)	Annual Tea Infusion Intake (L) *	Daily Tea Infusion Intake (L) *
19–29	480	48	0.132
30–59	480	48	0.132
60–74	600	60	0.164
≥75	720	72	0.197

* Assuming that 2 g of tea yield 200 mL of tea infusion.

## Data Availability

The original contributions presented in this study are included in the article/Supplementary Materials. Further inquiries can be directed to the corresponding authors.

## Supplementary Materials

The following supporting information can be downloaded at: https://www.mdpi.com/article/10.3390/foods14172944/s1, Table S1: Detailed descriptive statistics of teas divided by their type; Table S2: Detailed descriptive statistics of teas divided by their origin; The Target Hazard Quotient (THQ) Calculator.
