# Correction: Małyszek et al. Assessment of Fluoride Intake Risk via Infusions of Commercial Leaf Teas Available in Poland Using the Target Hazard Quotient Index Approach. *Foods* 2025, *14*, 2944

**DOI:** 10.3390/foods14193322

**Published:** 2025-09-25

**Authors:** Agata Małyszek, Ireneusz Zawiślak, Michał Kulus, Adam Watras, Julia Kensy, Agnieszka Kotela, Marzena Styczyńska, Maciej Janeczek, Jacek Matys, Maciej Dobrzyński

**Affiliations:** 1 Department of Biostructure and Animal Physiology, Wrocław University of Environmental and Life Sciences, Kożuchowska 1, 51-631 Wrocław, Poland; maciej.janeczek@upwr.edu.pl; 2 Department of Human Nutrition, Wrocław University of Environmental and Life Sciences, Chełmońskiego 37/41, 51-630 Wrocław, Poland; ireneusz.zawislak@upwr.edu.pl (I.Z.); marzena.styczynska@upwr.edu.pl (M.S.); 3 Division of Ultrastructural Research, Wrocław Medical University, Chałubińskiego 6a, 50-368 Wrocław, Poland; michal.kulus@umw.edu.pl; 4 Department of Pediatric Dentistry and Preclinical Dentistry, Wrocław Medical University, Krakowska 26, 50-425 Wrocław, Poland or a.watras@intibs.pl (A.W.); maciej.dobrzynski@umw.edu.pl (M.D.); 5 Institute of Low Temperatures and Structure Research, Polish Academy of Sciences, Okólna 2, 50-422 Wrocław, Poland; 6 Faculty of Dentistry, Wrocław Medical University, Krakowska 26, 50-425 Wrocław, Poland; julia.kensy@student.umw.edu.pl; 7 Medical Center of Innovation, Wrocław Medical University, Krakowska 26, 50-425 Wrocław, Poland; kotela.agnieszka@gmail.com; 8 Dental Surgery Department, Wrocław Medical University, Krakowska 26, 50-425 Wrocław, Poland

**Missing Supplementary File**

In the original publication [1], a supplementary file, “The Target Hazard Quotient (THQ) Calculator”, was not published. This file has now been added to the online version of the article and is listed in the Supplementary Materials Section.

**Text Correction**

Due to the updated supplementary files, references had to be revised accordingly. A correction has been made to Sections 3.1 and 3.4:

“Detailed descriptive statistics, including medians, interquartile ranges, and outliers, are provided in Supplementary Tables S1 and S2.”

“the calculator calculates the THQ value and informs the user whether there is a non-carcinogenic health risk associated with oral consumption of fluoride with tea infusion (THQ ≥ 1) or not (THQ < 1) (see ‘Supplementary Materials: THQ Calculator’).”

**Supplementary Materials**

Readability of the Supplementary Tables S1 and S2 has been corrected. Additionally, units have been added to the heading of Supplementary Table S2 as they have been initially omitted.

**References:**

In references [72,74] of the original publication, “PH” has been corrected to “pH”.

The authors state that the scientific conclusions are unaffected. This correction was approved by the Academic Editor. The original publication has also been updated. 
