# Supplementary material for: Assessment of Fluoride Intake Risk via Infusions of Commercial Leaf Teas Available in Poland Using the Target Hazard Quotient Index Approach
Source: Foods. 2025 Aug 24;14(17):2944. doi: 10.3390/foods14172944 (PMC12428000; doi:10.3390/foods14172944)
Supplement: Supplementary file 1 [file foods-14-02944-s001.zip › foods-3829356-supplementary.pdf]

Table S1. Detailed descriptive statistics of teas divided by their type.

	Type	pH	F [ppm]	P inorganic [ppm]	Ca [ppm]	buffer capacity [mM/l]	titratable acidity [mM/l]
**N**	**black**	55	55	55	55	55	55
	**oolong**	9	9	9	9	9	9
	**green**	27	27	27	27	27	27
	**white**	7	7	7	7	7	7
**Mean**	**black**	4.75	0.319	21.1	30.0	0.703	1.45
	**oolong**	4.98	0.324	10.6	23.9	0.309	0.700
	**green**	5.44	0.353	13.1	23.9	0.429	0.828
	**white**	5.39	0.217	12.4	25.4	0.305	0.536
**Median**	**black**	4.63	0.259	18.7	26.9	0.588	1.40
	**oolong**	4.97	0.200	6.35	22.0	0.256	0.560
	**green**	5.48	0.302	10.7	20.6	0.364	0.800
	**white**	5.40	0.240	10.2	20.6	0.282	0.640
**Standard deviation**	**black**	0.316	0.254	9.09	15.3	0.378	0.532
	**oolong**	0.375	0.359	9.25	8.63	0.123	0.390
	**green**	0.349	0.259	8.54	13.4	0.236	0.388
	**white**	0.424	0.153	5.83	17.6	0.139	0.223
**Minimum**	**black**	4.44	0.0470	3.96	3.25	0.111	0.220
	**oolong**	4.55	0.100	2.82	12.3	0.150	0.300
	**green**	4.66	0.0600	2.17	7.82	0.146	0.200
	**white**	4.53	0.0670	7.80	8.46	0.131	0.220
**Maximum**	**black**	5.67	1.52	45.5	67.7	2.00	3.40
	**oolong**	5.61	1.26	31.0	36.9	0.540	1.56
	**green**	6.09	1.23	33.4	63.7	1.33	1.80
	**white**	5.84	0.502	24.8	59.3	0.500	0.750

Table S2. Detailed descriptive statistics of teas divided by their origin.

Descriptives

	Region	pH	F [ppm]	P inorganic [ppm]	Ca [ppm]	buffer capacity [mM/l]	titratable acidity [mM/l]
**N**	**Africa**	8	8	8	8	8	8
	**Central Asia**	50	50	50	50	50	50
	**East Asia**	40	40	40	40	40	40
**Mean**	**Africa**	4.84	0.659	21.8	28.3	0.560	1.63
	**Central Asia**	4.91	0.285	19.0	27.4	0.613	1.23
	**East Asia**	5.17	0.300	14.4	27.2	0.501	0.943
**Median**	**Africa**	4.62	0.289	20.0	22.6	0.551	1.40
	**Central Asia**	4.67	0.259	17.3	24.2	0.528	1.25
	**East Asia**	5.30	0.261	11.7	23.9	0.421	0.800
**Standard deviation**	**Africa**	0.404	0.555	11.6	19.5	0.157	0.715
	**Central Asia**	0.421	0.208	9.45	12.5	0.378	0.493
	**East Asia**	0.480	0.177	8.98	16.3	0.341	0.593
**Minimum**	**Africa**	4.54	0.224	8.11	8.90	0.351	0.700
	**Central Asia**	4.45	0.0470	2.17	3.25	0.111	0.220
	**East Asia**	4.44	0.0700	2.17	7.82	0.131	0.200
**Maximum**	**Africa**	5.65	1.52	44.6	60.9	0.830	2.80
	**Central Asia**	6.02	1.26	45.5	59.3	2.00	2.60
	**East Asia**	6.09	1.05	33.4	67.7	1.67	3.40
